# A Viral microRNA Cluster Strongly Potentiates the Transforming Properties of a Human Herpesvirus

**DOI:** 10.1371/journal.ppat.1001294

**Published:** 2011-02-17

**Authors:** Regina Feederle, Sarah D. Linnstaedt, Helmut Bannert, Helge Lips, Maja Bencun, Bryan R. Cullen, Henri-Jacques Delecluse

**Affiliations:** 1 Department of Virus Associated Tumours, German Cancer Research Center, Heidelberg, Germany; 2 Center for Virology and Department of Molecular Genetics and Microbiology, Duke University Medical Center, Durham, North Carolina, United States of America; University of North Carolina at Chapel Hill, United States of America

## Abstract

Epstein-Barr virus (EBV), an oncogenic human herpesvirus, induces cell proliferation after infection of resting B lymphocytes, its reservoir *in vivo*. The viral latent proteins are necessary for permanent B cell growth, but it is unknown whether they are sufficient. EBV was recently found to encode microRNAs (miRNAs) that are expressed in infected B cells and in some EBV-associated lymphomas. EBV miRNAs are grouped into two clusters located either adjacent to the BHRF1 gene or in introns contained within the viral BART transcripts. To understand the role of the BHRF1 miRNA cluster, we have constructed a virus mutant that lacks all its three members (Δ123) and a revertant virus. Here we show that the B cell transforming capacity of the Δ123 EBV mutant is reduced by more than 20-fold, relative to wild type or revertant viruses. B cells exposed to the knock-out virus displayed slower growth, and exhibited a two-fold reduction in the percentage of cells entering the cell cycle S phase. Furthermore, they displayed higher latent gene expression levels and latent protein production than their wild type counterparts. Therefore, the BHRF1 miRNAs accelerate B cell expansion at lower latent gene expression levels. Thus, this miRNA cluster simultaneously enhances expansion of the virus reservoir and reduces the viral antigenic load, two features that have the potential to facilitate persistence of the virus in the infected host. Thus, the EBV BHRF1 miRNAs may represent new therapeutic targets for the treatment of some EBV-associated lymphomas.

## Author Summary

To persist in their hosts, herpes viruses must avoid recognition by the host's immune system. Down-regulation of viral antigen production through expression of viral miRNAs is a particularly elegant mechanism as these genetic elements do not encode proteins and remain therefore invisible to the immune system. Upon primary infection, Epstein-Barr virus (EBV) colonizes B cells and, through expression of its latent proteins, induces their continuous proliferation. The resulting expansion of infected B cells elicits a T cell response directed against the latent proteins that results in their elimination. Therefore, rapid proliferation of infected B cells, combined with reduced latent protein production, would facilitate establishment of EBV's viral reservoir before mounting of the immune response. Here, we find that a cluster of three microRNAs encoded near the EBV BHRF1 gene is crucial for efficient B cell transformation. In the absence of these genetic elements, infected B cells grow markedly more slowly. Furthermore, B cells exposed to an EBV mutant that lacks the BHRF1 microRNA cluster produced more latent proteins. Thus, the BHRF1 microRNA cluster possesses properties that potentiate EBV's oncogenic properties and therefore facilitate expansion of the EBV B cell reservoir.

## Introduction

Epstein-Barr virus (EBV) establishes a clinically silent chronic infection in the large majority of the world population [1]. In the course of primary infection, B cells targeted by the virus initiate transient cell growth until EBV-specific CD8+ T cells mount an efficient antiviral response [2]. However, some EBV-infected B cells will persist in the infected individual and form a long-lasting virus reservoir. In transplant recipients undergoing immunosuppressive therapy, EBV-induced B cell growth can lead to the development of a post-transplant lymphoproliferative disorder (PTLD) [1], and analogous conditions have been observed in other immunodeficient patients. This process can be reproduced *in vitro*; exposure of resting B cells to the virus leads to the generation of continuously growing lymphoblastoid cell lines (LCL). Both typical PTLD cells and LCLs produce all six Epstein-Barr virus nuclear antigens (EBNA) and three latent membrane proteins (LMP), collectively designated latent proteins [1]. Viral mutants that lack EBNA1, EBNA2, EBNA-LP or LMP1 display a massive reduction in their ability to transform B cells [3]–[7], thereby demonstrating that latent proteins are necessary for this process. However, it remains unclear whether they are sufficient for Bcell transformation.

**Figure 1:**
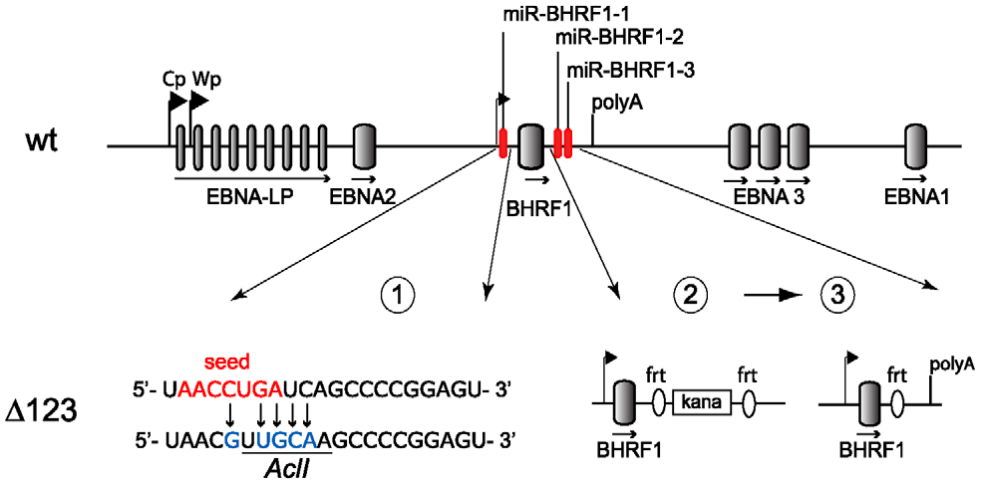
Construction of the BHRF1 miRNA viral mutant. Schematic representing part of the EBV wild type genome with a focus on the BHRF1 miRNA cluster (top panel; not to scale). The two promoters for latent EBNA transcripts (Cp, Wp) and the polyA site of the BHRF1 gene are indicated. Construction of the Δ123 mutant was performed in three sequential steps (lower panel). First, the seed region of miR-BHRF1-1 was mutated using chromosomal building. The seed region is indicated in red and introduced sequence changes are shown in blue. This also created a new AclI restriction enzyme site (underlined). Second, the mature miR-BHRF1-2 and miR-BHRF1-3 sequences were replaced by a kanamycin resistance cassette flanked by flip recombinase target (frt) sites. Third, transient expression of the flp recombinase led to the excision of the kanamycin cassette.

The EBV genome encodes a large number of non-coding RNAs that include the Epstein-Barr encoded RNAs (EBERs), as well as 25 miRNAs and one small nucleolar RNA (snoRNA) [8]–[12]. MiRNAs bind to mRNAs that contain fully or partially complementary sequences and as a consequence usually impair their translation and reduce their stability [13]. The EBV miRNAs are distributed in two clusters, located in the BART region or within the BHRF1 gene locus [9], [10]. The latter cluster comprises three members, two of which are located in the BHRF1 3′ untranslated region and the third immediately 5′ to the BHRF1 lytic mRNA transcription start site [9], [10] (Fig. 1A). They are processed from introns located within ∼100-kb long RNA transcripts that initiate at the Cp or Wp promoters used by all EBNA genes [14]. Expression of the BHRF1 miRNAs is characteristic of EBV-transformed B cells that express all latent genes (latency III) [1], [10]. In contrast, Bcell tumours such as Burkitt's lymphomas or epithelial cell tumours that express a restricted number of latent proteins (latency I or II) do not express the BHRF1 miRNAs [10]. However, induction of virus replication in some Burkitt's lymphoma cells leads to re-expression of the miRNA BHRF1 cluster, suggesting that these miRNAs may serve functions not only during latency III but also upon induction of virus replication [15]. In an attempt to understand the function of the BHRF1 miRNA cluster in continuously growing lymphoblastoid B cell lines, we constructed viruses that lack these miRNAs and report here their phenotypic traits.

## Results

### Construction of viral mutants

**Figure 2:**
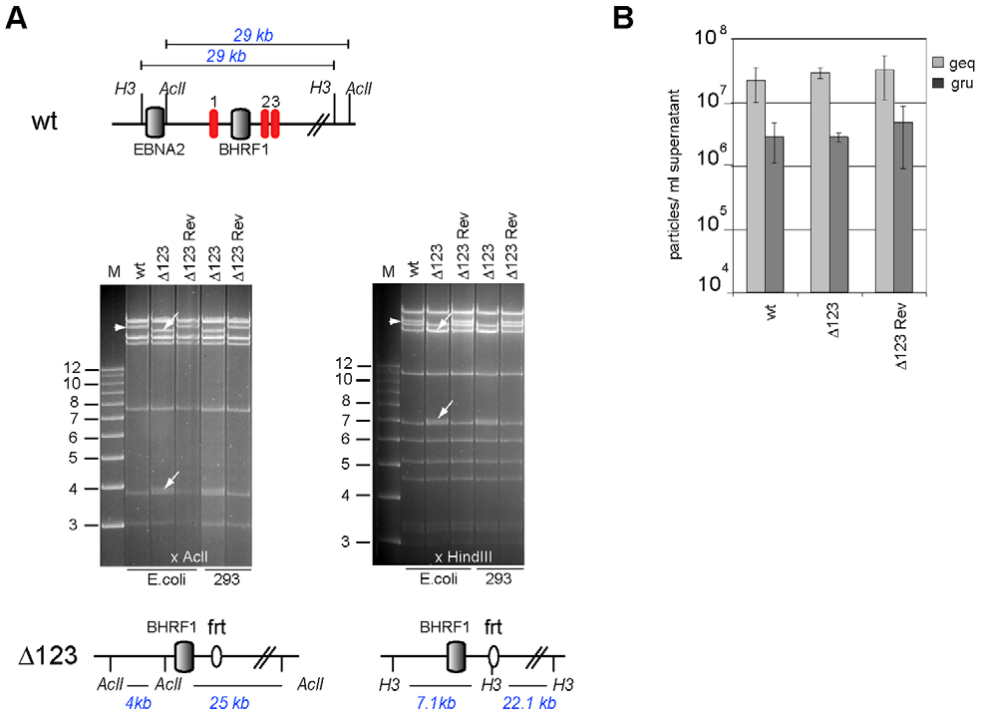
Characterization of viral recombinants. (A) EBV-wt, Δ123, and Δ123 Rev restriction enzyme analysis. A schematic map of the EBV-wt genome segment encompassing the BHRF1 region with cleavage sites for AclI and HindIII and the expected fragment sizes are shown on the top panel. The recombinant viral genomes isolated either from *E.coli* cells or rescued from stably transfected HEK293 cells were cleaved with AclI (left) or HindIII (right). While the exchange of miR-BHRF1-2 and miR-BHRF1-3 against a frt-site introduced a HindIII restriction site, the mutation of miR-BHRF1-1 introduced an AclI restriction site. The fragment size predictions for each enzyme are given and the resulting fragment changes observed after restriction analysis are indicated with arrows (lower panel). H3: HindIII; M: molecular weight marker; frt: flp recombinase target site. (B) Viral genome DNA equivalents (geq) per ml of supernatant were quantified by qPCR amplification of the viral BALF5 gene. Concentration of infectious particles per ml of supernatant were calculated by counting gfp-positive Raji units (gru). Mean values of 3 different supernatants are presented.

We deleted the BHRF1 miRNA cluster from the B95.8 genome cloned in *E.coli* in a sequential manner (Fig. 1). We first exchanged the miR-BHRF1-1 seed region for an unrelated sequence by chromosomal building using a shuttle plasmid that carries an ampicillin resistance cassette [16]. The modified miR-BHRF1-1 seed region introduces an AclI restriction site that allows unequivocal identification of the properly recombined mutant. In a second step, we replaced the DNA region that spans the miR-BHRF1-2 and miR-BHRF1-3 mature miRNAs by a kanamycin resistance cassette flanked by Flp recombinase target sites using recA-mediated homologous recombination. The last step consisted in excising the kanamycin resistance cassette by transient expression of the Flp recombinase. As a result, the miR-BHRF1-2 and miR-BHRF1-3 miRNAs were exchanged against one Flp recombinase target site (Fig. 1 and Fig. S1). The modified viral DNA, which carries a hygromycin resistance cassette, hereinafter referred to as Δ123, was then transfected into 293 cells. Clones from these stably transfected 293 cells (293/Δ123) were obtained by hygromycin selection and the viral mutant genomes present in these producing cell lines were transferred back into *E.coli* and their global integrity was confirmed by restriction enzyme analysis (Fig. 2A). Furthermore, sequencing the DNA fragments that were modified during virus construction confirmed the exactitude of the introduced alterations (Fig. S2) and the complete identity of sequences outside the miRNAs with wild type genome. Next, the producer cell clones were tested for their ability to sustain viral lytic replication. The viral structural titers were detected by quantitative PCR and found to be similar to those observed with wild type producer cell lines. The mean values ranged between 2.2×10^7^ and 2.9×10^7^ genome equivalents per ml of supernatant for Δ123 and wt, respectively, showing that the BHRF1 miRNAs are not required for virus production (Fig. 2B). We then incubated Raji B cells with these supernatants at various dilutions. Three days later the number of gfp-positive Raji cells was determined to assess functional infectious titers (Fig. 2B). The ratio between structural titers (geq/ml) and functional titers (gru/ml) was found to be 7.8 and 10.3 for wt and Δ123 viruses, respectively. We therefore conclude, that the BHRF1 miRNAs are not essential for EBV infection but we cannot rule out a minor contribution to this process.

The genome with the triple miRNA mutation formed the basis for construction of a revertant virus in which the modified sequences were re-exchanged with the original ones by chromosomal building to generate a Δ123 revertant (Δ123 Rev) virus genome [16]. This technique allows exact reconstruction of the original wild type sequence. The reverted genome was introduced in turn into 293 cells from which hygromycin-resistant clones were selected. Restriction analysis and sequencing confirmed that the revertant virus shared perfect homology with the wild type EBV genome (Fig. 2A and Fig. S2). Producer clones carrying the revertant genome delivered structural and functional titers akin to those observed with wt viruses (Fig. 2B).

### A virus that lacks the BHRF1 miRNA cluster displays reduced transformation capacity

**Figure 3:**
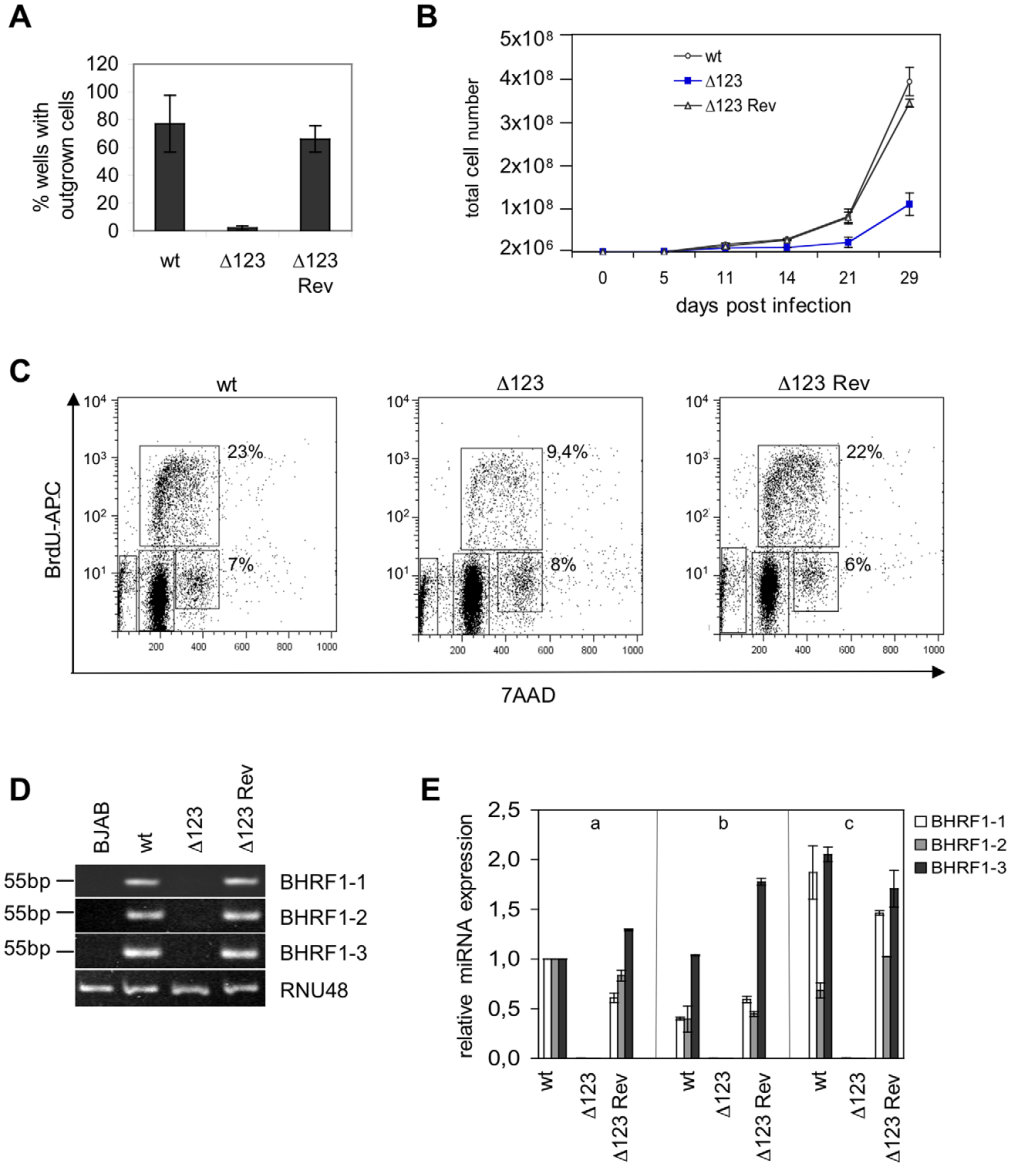
B cells transformed by Δ123 virus show defective cell growth relative to its wild type counterparts. (A) B cell transformation assays were carried out at low B cell concentration (10^2^/well) with an MOI of 1 gru per B cell. An average of the results of three independent infection experiments is presented. (B) Growth curve of EBV-infected B cells in the first weeks post-infection. Mean values of three independent B cell infection experiments are shown. (C) Cell cycle analysis of BrdU-7AAD-stained LCLs generated with Δ123, Δ123 Rev, and EBV-wt viruses. The percentage of cells present in S or G2/M phase of the cell cycle is given. (D) BHRF1 miRNA expression profile. Shown are RT-PCR amplification products from one LCL set generated with Δ123, Δ123 Rev, or EBV-wt viruses using primers specific for miR-BHRF1-1, miR-BHRF1-2, and miR-BHRF1-3. Amplification of RNU48 served as an internal reference, and BJAB cells were used as a miRNA negative control. (E) Three unrelated B cell samples (a, b, and c) were infected with Δ123, Δ123 Rev, or EBV-wt viruses and BHRF1 miRNA expression was evaluated at day 11 p.i. Results are presented relative to the values obtained with one LCL (a) exposed to EBV-wt. The mean value of three independent analyses are shown.

To assess the contribution of the BHRF1 miRNA cluster to EBV's transforming properties, we exposed resting primary B cells to wild type, Δ123, and Δ123 Rev viruses. Infections were carried out at an MOI of 1 infectious particle per B cell (i.e. one gru/B cell), and cell outgrowth was monitored. Infected B cells were either seeded at low concentration, i.e. 2×10^3^ per ml in a 96-well plate containing feeder cells or at high concentration i.e. 2×10^6^ cells per ml. EBV-infected cells grow much more easily when infected at high concentration. Therefore, the first culture conditions are very stringent and allow detection of differences in terms of transformation efficiency but they do not allow monitoring of the infected B cells at the early stages of transformation. The percentage of wells containing outgrowing cell clones was assessed 8 weeks after infection. The results of three independent infection experiments is presented in Figure 3A. On average, wild type and revertant viruses respectively induced 82 and 75% cell outgrowth at an MOI of 1. In contrast, only 3% of the wells containing B cells infected with Δ123 virus showed outgrowth (Fig. 3A). Note that the standard variation between the different transformation assays was high. This reflects the fact that B cells from different individuals differ in their ability to form continuously growing cell lines. We conclude from these data that the BHRF1 miRNA cluster markedly increases the efficiency of transformation at low B cell concentration. The results of the bulk infection revealed similar though less pronounced effects. Monitoring of cell growth in B cells exposed to EBV-wt, Δ123, and Δ123 Rev virus evidenced slower growth in samples infected with Δ123. After 29 days in culture, B cells exposed to wild type or Δ123 Rev viruses expanded from 2×10^6^ to 3.6×10^8^ and 3.3×10^8^ cells, respectively. This compares with 1.3×10^8^ for B cells transformed by Δ123 (Fig. 3B). This prompted us to study the cell cycle parameters of B cell populations, by performing a BrdU incorporation assay (Fig. 3C). Whilst between 22 and 23% of B cells infected with both wild type controls entered S phase within 30 minutes, this percentage fell to 9.4% in B cells infected with Δ123 virus. We also noted that the ratio between cells still present in G2/M phase at the time of analysis and those that had entered S phase was ∼0.3 for the controls and ∼0.9 for B cells infected Δ123 virus. From this set of data, we conclude that the absence of BHRF1 miRNAs does not suppress EBV's transforming properties, but instead markedly slows down the growth rate of infected target cells. The permanently growing cell lines obtained after Δ123 infection provided material to quantify BHRF1 miRNA expression by RT-qPCR using the RNU48 snoRNA as an internal reference. The results of this analysis are given in Fig. 3D and show expression of the three BHRF1 miRNAs in all control lines tested, but not in those generated with Δ123, as expected. Of note, the expression level of the BHRF1 miRNAs varies within the control LCLs established from three different B cell infections (1 to 5 range for miR-BHRF1-1, 1 to 4 range for miR-BHRF1-2, 1 to 2 range for miR-BHRF1-3). This variation most likely reflects different activity levels of the Cp/Wp promoter among different LCLs [15].

### B cells infected with the BHRF1 miRNA triple mutant retain a high latent gene level of transcription and translation over time

**Figure 4:**
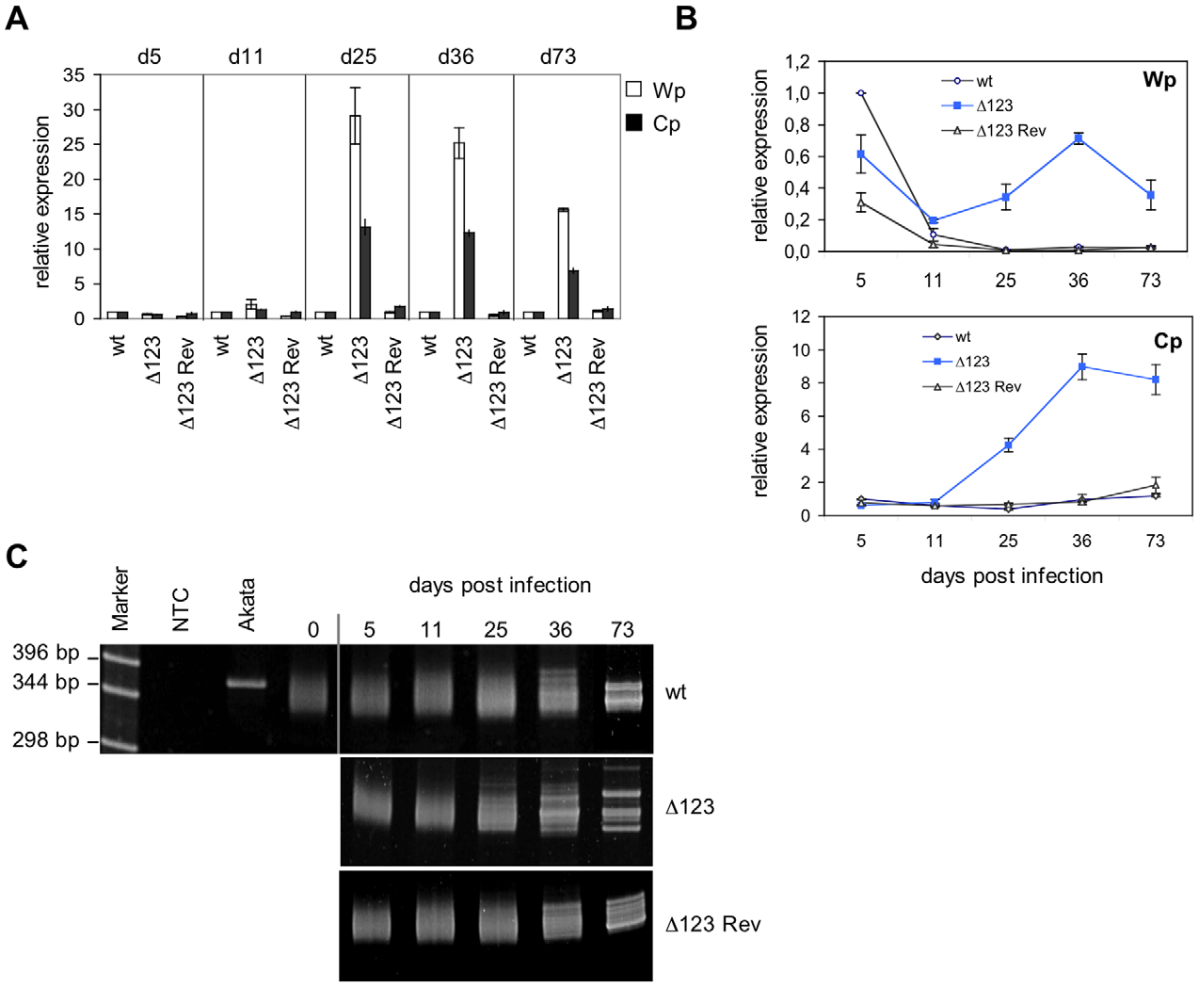
Latent promoter usage and clonality studies of transformed B cells. (A) Wp and Cp-initiated transcripts were monitored by RT-qPCR in B cells transformed with Δ123, Δ123 Rev, or EBV-wt virus at multiple time points post-infection. Results obtained at a given time point are grouped and presented relative to EBV-wt at the same time point. Results represent mean values obtained from three independent analyses. (B) The same data as in (A) presented relative to the value observed in B cells infected by EBV-wt five days after infection. This illustrates Wp and Cp activities over time in B cells infected by Δ123 mutant or wild type counterparts. (C) Clonality of LCLs generated with Δ123, Δ123 Rev, or EBV-wt virus was determined by a PCR-based assay using primers specific to the IgVH sequences. Cell populations were analyzed at different time points post-infection. Akata is a monoclonal B cell line; non-infected primary B cells (day 0) are polyclonal in nature. NTC: no template control.

The latent viral proteins have been recognized as the principal mediators of EBV's transforming properties. It was therefore important to assess latent gene expression in B cells infected with the EBV Δ123 mutant. To this aim, we carried out RT-qPCR analysis of the viral latent transcripts produced from the Wp and Cp promoters in transformed Bcell lines at 5, 11, 25, 36, and 73 days after infection (Fig. 4A). At day 5, the level of transcription from the Cp and Wp promoters was very similar in cells infected with Δ123 and in wild type controls. However, from day 11 on, the activity of the Wp promoter, and of the Cp promoter gradually increased in B cells transformed with the triple mutant relative to their control counterparts (Fig. 4A). Rather than an absolute increase of Wp transcriptional activity in Δ123-positive B cell lines, the observed differences could be ascribed to a stronger down-regulation of Wp in the controls (Fig. 4B).

These results suggested that the BHRF1 miRNA cluster, directly or indirectly, negatively regulates the expression of Wp/Cp-driven transcripts. Alternatively, these results could be accounted for by selection of a minor population of B cells that happens to express very high levels of EBV latent genes, provided that such an overexpression confers a growth advantage in LCLs infected by EBV Δ123 mutant. To test this hypothesis, we performed clonality studies of the LCLs at the different time points used in transcriptional studies reported above (Fig. 4C). To this aim, immunoglobulin heavy chain (IgH) genes were amplified by PCR using degenerated primers that bind to a large number of IgH variable gene families [17]. Whilst an IgH PCR performed on monoclonal B cell populations will reveal a unique band, it will yield a smear that ranges between approximately 330 and 350 bp if applied to a polyclonal B cell population as the length of different IgH family members slightly differs [17]. IgH PCR performed on LCLs generated with Δ123 or with wild type virus controls displayed a polyclonal pattern at day 0 (before infection), day 5, day 11, and day 25 post-infection. From day 36 on, discrete bands become visible against a polyclonal background, and at day 73 all three cell lines contained multiple dominant discrete clones, ie they became oligoclonal (Fig. 4C). From these results, we infer that early selection of dominant clones does not take place in the LCL infected by the EBV Δ123 mutant.

**Figure 5:**
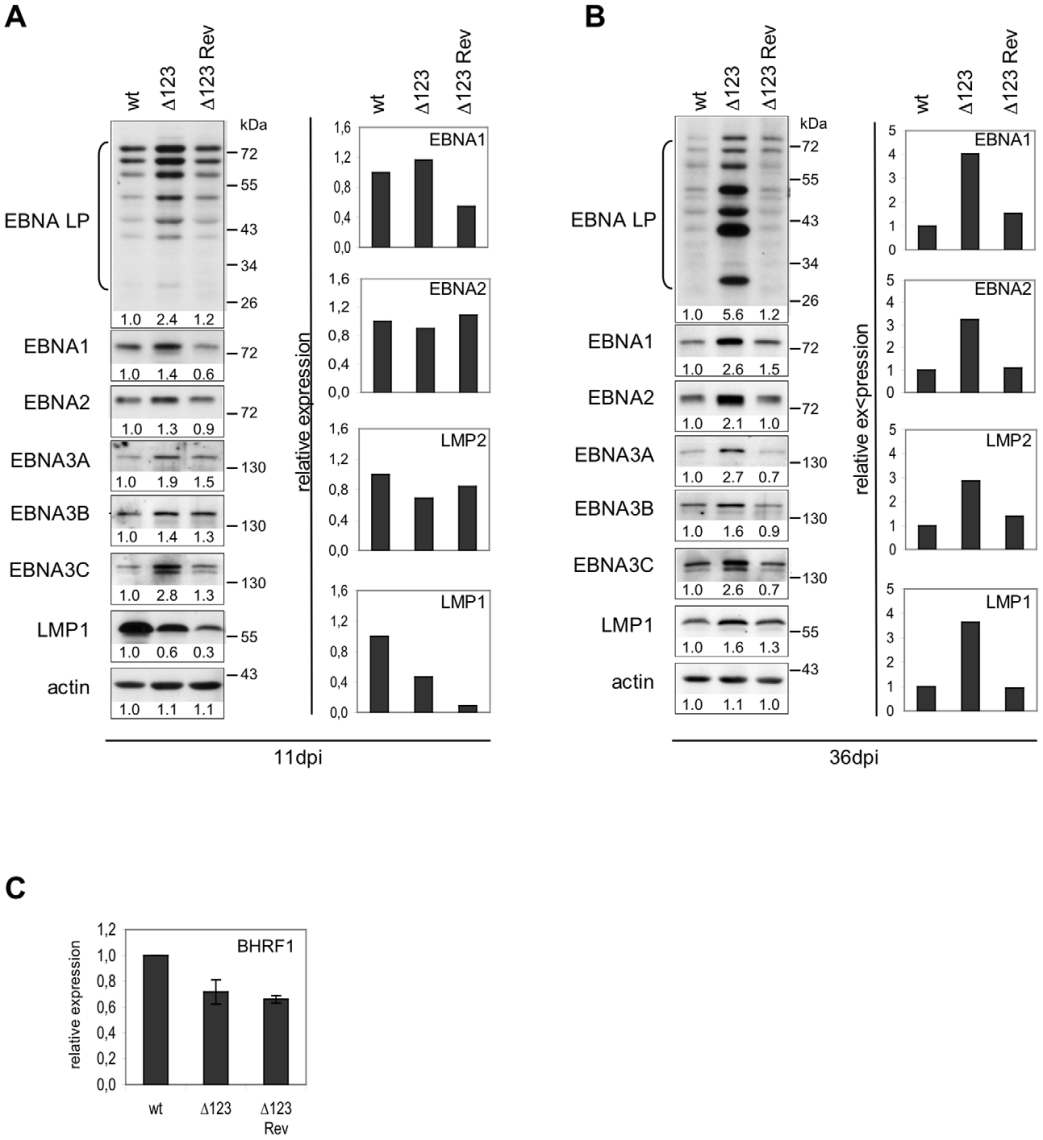
Latent gene expression profiles of B cells transformed byΔ123 virus. (A) and (B) EBV latent gene transcription and translation in LCLs transformed by Δ123, Δ123 Rev, or EBV-wt virus were examined by RT-qPCR (right panel) and western blot analysis (left panel) at day 11 (A) and day 36 (B) post-infection (dpi). Results from one representative experiment are presented. Intensity of western blot signals was measured using ImageJ software. Given below each western blot is the ratio between the intensity of the observed signal and the wt signal (wt set as 1). (C) BHRF1 gene transcription in LCLs transformed by Δ123, Δ123 Rev, or EBV-wt virus as measured by RT-qPCR. Data are means from three independent infection experiments.

We then used RT-qPCR and western blots to gauge EBV latent gene expression. The results of one out of four experiments are shown in Figure 5. This set of experiments demonstrated that B cells generated with the Δ123 virus express all tested latent genes with some expressed at higher levels than in the controls at day 11 post-infection (Fig. 5A). Similar assays conducted at day 36 post-infection confirmed this trend; all latent genes were expressed at higher levels in Δ123-positive LCLs both at the mRNA and the protein level (Fig. 5B). This difference was particularly dramatic in the case of the EBNA-LP protein, which was five times more abundant in LCLs generated with Δ123 than in controls, but was also visible for EBNA1, EBNA2, EBNA3A, EBNA3B, and EBNA3C. LMP1 and LMP2-specific transcripts were also more abundant in LCLs infected by the triple mutant. Western blot analysis detected a marginal increase in LMP1 protein production in the same cells. Recent recognition that the BHRF1 protein is important for B cell transformation prompted us to assess expression of this gene [18]. Lymphoblastoid cell lines infected with wt, Δ123, or Δ123 Rev viruses evinced similar levels of BHRF1 transcripts (Fig. 5C).

## Discussion

Since their initial discovery in the EBV genome, miRNAs have been found in all investigated herpesviruses [19], [20]. Although some of these miRNAs moderate the expression of virus replication proteins, e.g. miR BART2 and the EBV viral polymerase BART5, miR-H2, and the ICP0 transactivator, many have been found to regulate virus-cell interactions [21], [22]. Maintenance of latency appears to be a recurrent theme across herpes viruses [20], [23]. Examples are provided by HSV1 miR-M2-3p that inhibits translation of ICP0, a key transactivator that initiates virus lytic replication [22]. More recently, a cluster of 12 pre-miRNAs from the KHSV genome has been implicated in the repression of lytic replication through its ability to down-regulate IκBα [24]. Facilitation of immune evasion appears to be another crucial function served by several virus miRNAs [20]. Indeed, miR-UL112-1 from HCMV curbs synthesis of the NK cellular cell surface receptor MICB and EBV miR-BHRF1-3 prevents production of the putative T cell chemoattractant CXCL11 [25], [26]. In the present paper, we expand the spectrum of functions served by viral miRNAs during virus latency. We provide clear evidence that the BHRF1 miRNA cluster substantially enhances EBV's transforming potential. In its absence, B cells infected with the same functional MOI grew more slowly and could not efficiently form colonies at low concentration. Although we usually see EBV-mediated transformation as the molecular mechanism that underlies EBV's oncogenic properties, it is also activated during primary infection, presumably to expand the size of the Bcell reservoir. Our observations suggest that in the absence of the BHRF1 miRNA cluster, expansion of this compartment would be substantially reduced.

Importantly, the Δ123 defective phenotype could not be ascribed to a down-regulation of latent protein expression. This family of proteins has been found to activate several signal transduction pathways that include the Notch, NFkB, Jun, AP1, and JAK-Stat pathways and is therefore thought to be the principal effector of B cell transformation [14]. Although the level of latent transcription was nearly identical in B cells infected with Δ123 or in controls at day 5 post-infection, a divergence between both virus types became discernible after 11 days and became obvious after 3 weeks in culture. While B cells transformed with wild type viruses progressively but efficiently down-regulated Wp transcription starting at five days after infection, this process was much less efficient in cells transformed with Δ123 virus. In this respect, It is interesting to note that the kinetics of expression of the BHRF1 miRNA cluster and of Wp transcripts are exactly opposite; while miR-BHRF1-3 expression starts at day one and linearly increases until day 8, Wp transcripts are maximal at day 1 and sharply decrease until day 8 after which they more slowly reach a minimum plateau at day 20 [27], [28]. However, B cells infected by the Δ123 virus mutant also exhibited an increase in the abundance of Cp-initiated transcripts as soon as this promoter became dominant, three weeks after infection. Therefore, the deletion of the BHRF1 miRNA cluster prevents repression of the Wp transcripts and enhances expression of the Cp promoter.

This could suggest that the latent transcripts, from which the latent genes originate, are direct targets of the miRNA BHRF1 cluster. However, the high amplitude of the effects caused by the absence of the BHRF1 miRNAs is not consonant with those usually observed with viral or cellular miRNAs, even if we hypothesize a synergistic effect of the three miRNAs. Rather, they suggest that the BHRF1 cluster may target one or more cellular transactivators, many of which (BSAP/pax5, RFX1, YY1, MIBP1, CREB, ATF1) have been shown to regulate expression of the Wp/Cp promoters [14]. Using the photoactivatable ribonucleoside-enhanced crosslinking and immunoprecipitation (PAR-CLIP) technique recently described by the Tuschl laboratory [29], we have comprehensively identified mRNAs bound by all of the viral and cellular miRNAs expressed in LCLs (R. Skalsky and B. Cullen, manuscript in preparation). Using this technology, we have identified ∼300 mRNA targets for the EBV BHRF1 miRNAs. Although strong binding sites for cellular miRNAs on the viral LMP1 and BHRF1 mRNAs were readily detected, none of the latent EBV mRNAs were bound by any of the BHRF1 miRNAs. We therefore conclude that the observed effect of these viral miRNAs on EBV latent gene expression is indirect.

The enhanced latent transcription observed in B cells infected with Δ123 virus correlated with an increased production of latent proteins relative to wild type controls. This effect was most visible for EBNA-LP, but EBNA1, EBNA2, EBNA3A, EBNA3B, EBNA3C, and LMP2A were all more strongly expressed in B cells infected with the triple miRNA mutant. Therefore, the total amount of viral antigens present in B cells transformed by Δ123 is substantially higher than seen in wild type controls. Latent proteins, and in particular EBNA1, EBNA3A, and EBNA3C, have been shown to elicit a strong CD8+ T-cell response directed against EBV-infected cells. We infer from these data that the BHRF1 cluster reduces the antigenic load present in B cells and could therefore facilitate viral immune evasion [2] and thus persistence, a common trait of herpes viruses [20].

Is there a causal relationship between the relative excess of latent proteins observed in B cells transformed by Δ123 and the reduced growth rate? An excess of LMP1 has been reported to exert toxic effects on infected B cells [3], but the expression level obtained from the expression plasmid used in this work appears much higher than observed in Δ123-transformed LCLs. We therefore favor the view that the excess of latent genes cannot fully explain the Δ123 phenotype and that the BHRF1 cluster also acts independently of the viral latent genes, probably by targeting cellular genes involved in cell growth control.

Our data clearly document that the BHRF1 miRNA cluster serves a central function in EBV biology as it enhances the virus' ability to dysregulate B cell growth. Thus, the miRNA cluster expands the size of EBV's reservoir and enhances its oncogenic property. Therefore, it represents a new potential therapeutic target for the treatment of EBV-associated diseases.

## Materials and Methods

### Primary cells and cell lines

HEK293 cells are neuro-endocrine cells obtained by transformation of embryonic epithelial kidney cells with adenovirus [30], [31]. Raji and Akata are EBV-positive Burkitt's lymphoma cell lines, BJAB is an EBV-negative Burkitt's lymphoma cell line [32]–[34]. WI38 are primary human lung embryonic fibroblasts [35]. All cell lines were grown in RPMI 1640 medium supplemented with 10% fetal calf serum (FCS; Biochrom).

### Plasmids

We introduced mutations in miR-BHRF1-1 (B95.8 co-ordinates 53762-53782; accession number V01555.2) by overlap PCR using mutated primers (TAACCTGATCAGCCCC changed into TAACGTTGCAAGCCCC). This gave rise to a 1.8 kb fragment (B95.8 coordinates 52807-54651) that encompasses a BHRF1-1 miRNA with a mutated seed region. Furthermore, the introduced mutation creates an AclI restriction site that allows distinction between the wild type and the mutated sequences (see also Fig. 1). The 1.8 kb PCR fragment was cloned into a plasmid (B269) that consists of an arabinose-inducible temperature sensitive bacterial origin of replication, the ampicillin (amp) resistance gene, RecA and the LacZ operon to generate a shuttle vector (B396) for chromosomal building. A 2.9 kb BspT1/SalI fragment (B95.8 coordinates 53222-56081) was cloned into the vector B269 to generate the shuttle vector for construction of the Δ 23 revertant (B 430).

### Construction of viral mutant and revertant

The wild-type EBV B95.8 strain cloned onto a prokaryotic F-plasmid, which carries the chloramphenicol (cam) resistance gene, the gene for the green fluorescent protein (GFP), and the hygromycin (hyg) resistance gene (p2089) [36], was used to generate the BHRF1 miRNA mutant. Deletion of miR-BHRF1-1 was performed by chromosomal building as described [16] using B396 as targeting vector. This involved initial building of a cointegrate between the shuttle vector that contains the mutated version of the mature miR-BHRF1-1 and the wild type EBV genome. This co-integrate that as a result of recombination carries both mutated and wild type miR-BHRF1-1 was then resolved to eliminate the wild type copy of the miRNA. Subsequently, a triple mutant lacking all three BHRF1 miRNAs was obtained by exchanging the viral DNA fragment that contains both mature miR-BHRF1-2 and miR-BHRF1-3 and the sequence between them (B95.8 co-ordinates 55176-55279) against the kanamycin resistance gene flanked by flp-recombination sites as described [16]. The kanamycin resistance cassette from pCP15 was amplified using primers 682 CTTTTAAATTCTGTTGCAGCAGATAGCTGAT ACCCAATGTAACAGCTATGACCATGATTACGCC and 683 ATTTTAACGAAGAGCGTGAAGCACCGCTTGCAAATTACGTCCAGTCACGACGTTGTAAAACGAC . The kanamycin cassette was excised from recombined clones by transient transformation of the Flp recombinase cloned onto a temperature-sensitive plasmid (pCP20). A revertant clone for Δ123 was constructed by chromosomal building using the shuttle vector described above.

### Stable clone selection and plasmid rescue in E.coli

Recombinant EBV plasmids were transfected into HEK293 cells by lipofection (Metafectene, Biontex) and selected for hygromycin resistance (100 µg/ml). Recombinant EBV genomes were purified from GFP-positive hygromycin-resistant cell clones as described [37] and electroporated into *E. coli* DH10B cells (1200 V, 25 mF, 200 Ω). The genetic integrity of the mutant or revertant EBV genomes stably introduced in HEK293 cell clones was assessed by restriction enzyme analysis and DNA sequencing of the BHRF1 gene region. The mutant producer cell clones were designated as 293/Δ123 and the revertant producer clones thereof as 293/Δ123 Rev.

### Virus induction and titer quantification

All described producer cell lines clones were lytically induced by transfection of a BZLF1 expression plasmid together with a BALF4 expression plasmid (3 µg each/6-well plate). Medium was changed at day 1 post-infection (p.i.), virus supernatant harvested at day 4 p.i., filtered through a 0.45 µm filter, and stored in aliquots at −80°C. Viral genome equivalents (geq) were determined by quantitative real-time PCR using EBV BALF5-specific primers and probe as described [38]. Infectious titers were determined by infecting 10^4^ Raji B cells with increasing 5-fold dilutions of supernatants. Three days after infection, gfp-positive cells were counted using a fluorescent microscope.

### Infection of primary B lymphocytes

Primary B cells were freshly isolated from adult human blood buffy coats by density gradient centrifugation followed by positive selection using M-450 CD19 Dynabeads (Dynal). To quantify EBV-mediated B cell transformation rates, freshly isolated primary B cells were exposed to infectious supernatants at a MOI of 1 infectious particle (gru) per B cell overnight at 37°C and then seeded at a concentration of 100 cells per well in 96-U-well plates coated with lethally irradiated WI38 feeder cells. The number of wells with proliferating cells was determined 8 weeks post-infection. Infection experiments were also carried out at high B cell concentration; 2x10e6 B cells were exposed to infectious supernatant at an MOI of 1 infectious particle per cell and were harvested for RNA and protein extraction at different time points post-infection.

### Stem-loop real-time PCR

BHRF1 miRNAs extracted from lymphoblastoid cell clones were reverse transcribed using specific stem-loop primers and TaqMan miRNA reverse Transcription Kit (Applied Biosystems) as described elsewhere [28], [39]. In brief, 110 ng of total RNA isolated with a miRNA extraction kit (Qiagen) was reverse transcribed as recommended by the manufacturer. The sequences of stem-loop primer, primer and probes and the qPCR conditions were identical to those described elsewhere [28]. Reverse transcription and amplification of cellular snoRNA RNU48 was performed in parallel to normalize for cDNA recovery (Assay ID 001006; Applied Biosystems) as recommended by the manufacturer. Real-time PCR was performed on an ABI 7300 Sequence Detection System (Applied Biosystems). All reactions were run in duplicates. An aliquot of the PCR reaction was taken after 30 cycles, products were loaded onto a 8% polyacrylamide gel and visualized by ethidium bromide staining.

### Western blot analysis

Cells were resuspended in PBS and lysed by sonication. 30 µg of proteins were denatured in Laemmli buffer for 5 minutes at 95°C, separated on a 10% or 7.5% SDS-polyacrylamide gel and electroblotted onto a Hybond ECL membrane (Amersham). After preincubation for 30 min in 5% milk powder in PBS, blots were incubated with primary antibodies EBNA1 (IH4), EBNA2 (PE2), LMP1 (S12), EBNA-LP (JF186), EBNA3A (ExAlpha), EBNA3B (ExAlpha), EBNA3C (A10), or actin (clone ACTN05; Dianova) for 1 h at room temperature. After several washings in 0.1% Tween in PBS, blots were incubated for 1 h with secondary antibodies coupled with horseradish peroxidase. Antibody binding was revealed using an ECL detection reagent (Perkin Elmer). Densitometric analyses of immunoblots were performed using ImageJ software.

### Real-time PCR of EBV latent genes

400 ng aliquots of RNA isolated from infected B cells (miRNeasy kit, Qiagen) was reverse transcribed with AMV-RT (Roche) using a mix of primers specific for various EBV transcripts and specific for GAPDH as described [18], [40]. Quantitative PCR using primers specific for Cp, Wp, LMP1, LMP2A, EBNA2, and YUK-spliced EBNA1 transcripts were performed as described [40]. PCR and data analysis was carried out using the universal thermal cycling protocol on an Applied Biosystem 7300 real-time PCR System. All samples were run in duplicates, together with primers for the amplification of the human GAPDH gene in combination with a VIC-labeled probe (Applied Biosystems) to normalize for variations in cDNA recovery.

### Cell cycle analysis

Cell cycle parameters of exponentially growing lymphoblastoid cell lines phase were monitored by exposing 1×10^6^cells for 30 minutes to BrdU (10 µM final concentration) (BrdU flow kit, BD Biosciences Pharmingen, San Diego, USA). The degree of incorporation was determined by immunostaining with an APC-coupled antibody directed against BrdU. DNA content was assessed by staining cells with the DNA intercalating dye 7-AAD. Analysis of fluorescence with a two laser FACScalibur flow cytometer allows identification of cells that have entered the S phase (BrdU positive), the G2/M phase (BrdU negative, double DNA content) or the G0/G1 phase (BrdU negative, double DNA content).

### PCR amplification of variable IgH sequences

DNA was extracted from 1 to 3×10^6^ cells using a DNeasy Tissue kit (Qiagen). IgH variable (IgVH) sequences spanning FR1, CDR1, FR2, CDR2, FR3, and CDR3 were PCR-amplified using a single consensus forward primer FR1c:5′AGGTGCAGCTGSWGSAGTCDGG 3′ and a mixture of JH family-specific reverse primers JH1/2/4/5 5′-ACCTGAGGAGACGGTGACCAGGGT-3′, JH3 5′- TACCTGAAGAGACGGTGACCATTGT-3′ and JH6 5′- ACCTGAGGAGACGGTGACCGTGGT-3′ described previously [17]. PCR amplification was performed in a reaction containing 1 µg heat denatured (10 min at 94°C) genomic DNA, 0.8 µM FR1c primer, 0.26 µM of each JH primer, 200 µM of each dNTP, 1.0 mM MgCl2, and 2.5 U Expand High Fidelity thermostable DNA polymerase (Roche). The first PCR cycle consisted of a denaturing step at 94°C for 2 min, followed by 30 cycles at 94°C for 30 s, at 61°C for 60 s, and at 72°C for 60 s (10 min in the last cycle). PCR products were then separated by electrophoresis on a 6% polyacrylamide gel, and bands corresponding to the 330–350 bp IgVH products visualized by ethidiumbromide staining.

## Supporting Information

Figure S1 Sequence of the BHRF1 region in wt and Δ 123 mutant. (A) EBV-wt sequence. Location of the BHRF1 intron (blue lettering), the BHRF1 open reading frame (green lettering) and the BHRF1 TATA box, the translation initiation site and poly A site (boxes) are indicated. The three BHRF1 pre-miRNAs are shown in red, the mature miRNAs in orange. (B) Δ 123 sequence. Transcripts are indicated in the same colors as described above. The miR-BHRF1-1 sequence was mutated at 5 positions (black lower case lettering). The region spanning the mature BHRF1-2 and BHRF1-3 miRNAs were replaced by a frt recombination site (boxed) and additional flanking sequences from the targeting vector. Corresponding EBV coordinates refer to the EBV B95.8 stain (accession number V01555.2).
(2.62 MB TIF)

Figure S2 Electropherogram of sequenced miRNA regions in the Δ 123 mutant and Δ 123 revertant. (A) Shown is the sequenced lower strand spanning miR-BHRF1-1 (underlined) from B95.8 coordinates 53823 to 53701 (accession number V01555.2). The seed region of BHRF1-1 is boxed, the introduced mutations in the Δ 123 mutant (upper panel) are highlighted by arrows. The lower panel shows that the same DNA region in the Δ 123 revertant is identical to the wt miR-BHRF1-1 sequence. (B) DNA fragment spanning the BHRF1-2 and BHRF1-3 miRNAs. Upper panel: In the Δ 123 mutant, the BHRF1-2 and BHRF1-3 miRNAs are replaced by sequences from the kanamycin targeting plasmid (underlined in orange) including one frt site (boxed). EBV-specific sequences are underlined in black. Lower panel: Sequence spanning miR-BHRF1-2 and miR-BHRF1-3 (underlined) in the Δ 123 revertant shows perfect homology to EBV-wt sequences. B95.8 coordinates are indicated.
(2.76 MB TIF)
