# Supplementary figures and images for: A Viral microRNA Cluster Strongly Potentiates the Transforming Properties of a Human Herpesvirus
Source: PLoS Pathog. 2011 Feb 17;7(2):e1001294. doi: 10.1371/journal.ppat.1001294 (PMC3040666; doi:10.1371/journal.ppat.1001294)

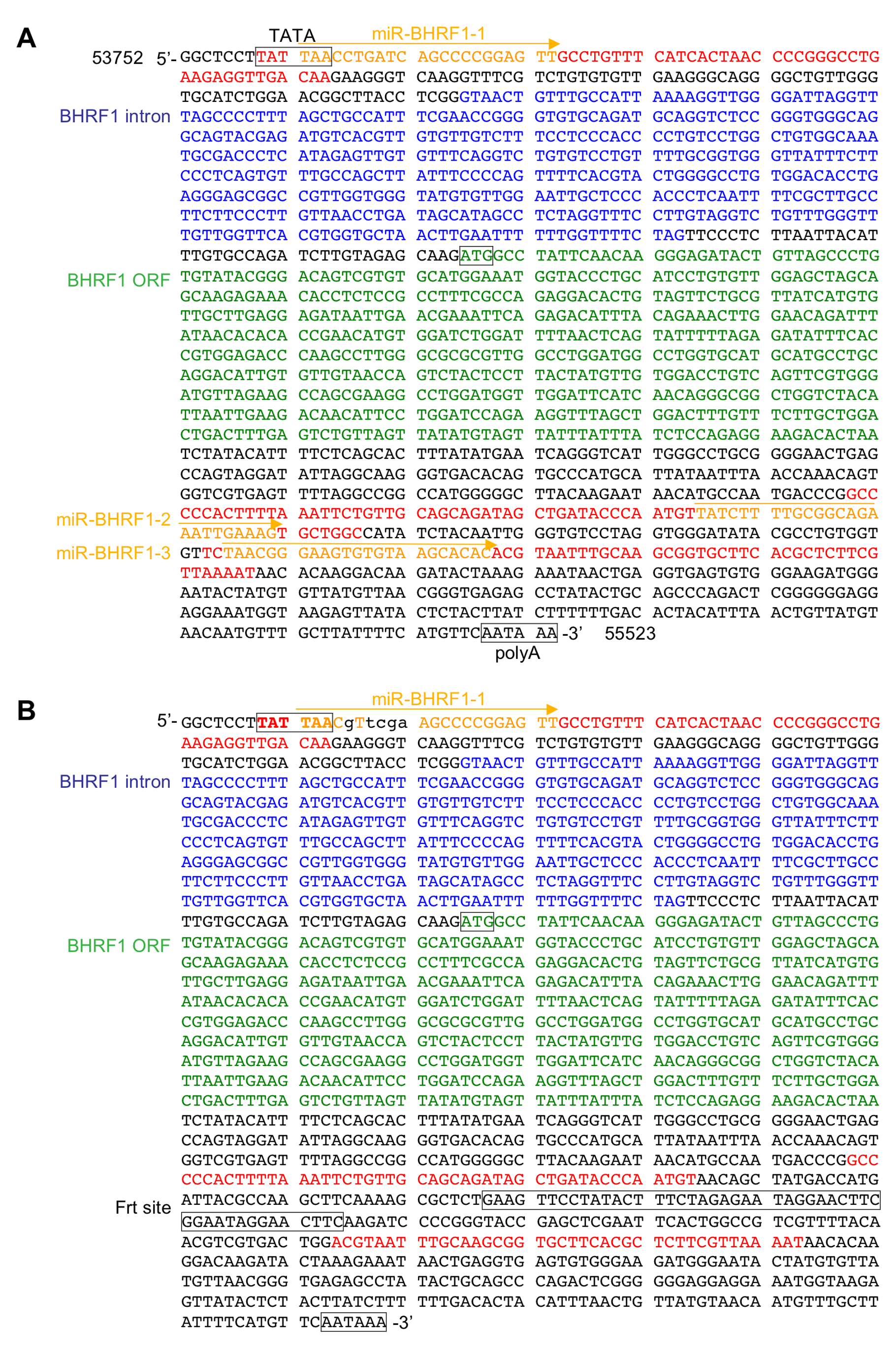

Supplement: Figure S1 — Sequence of the BHRF1 region in wt and Δ 123 mutant. (A) EBV-wt sequence. Location of the BHRF1 intron (blue lettering), the BHRF1 open reading frame (green lettering) and the BHRF1 TATA box, the translation initiation site and poly A site (boxes) are indicated. The three BHRF1 pre-miRNAs are shown in red, the mature miRNAs in orange. (B) Δ 123 sequence. Transcripts are indicated in the same colors as described above. The miR-BHRF1-1 sequence was mutated at 5 positions (black lower case lettering). The region spanning the mature BHRF1-2 and BHRF1-3 miRNAs were replaced by a frt recombination site (boxed) and additional flanking sequences from the targeting vector. Corresponding EBV coordinates refer to the EBV B95.8 stain (accession number V01555.2). (2.62 MB TIF) [file ppat.1001294.s001.tif]

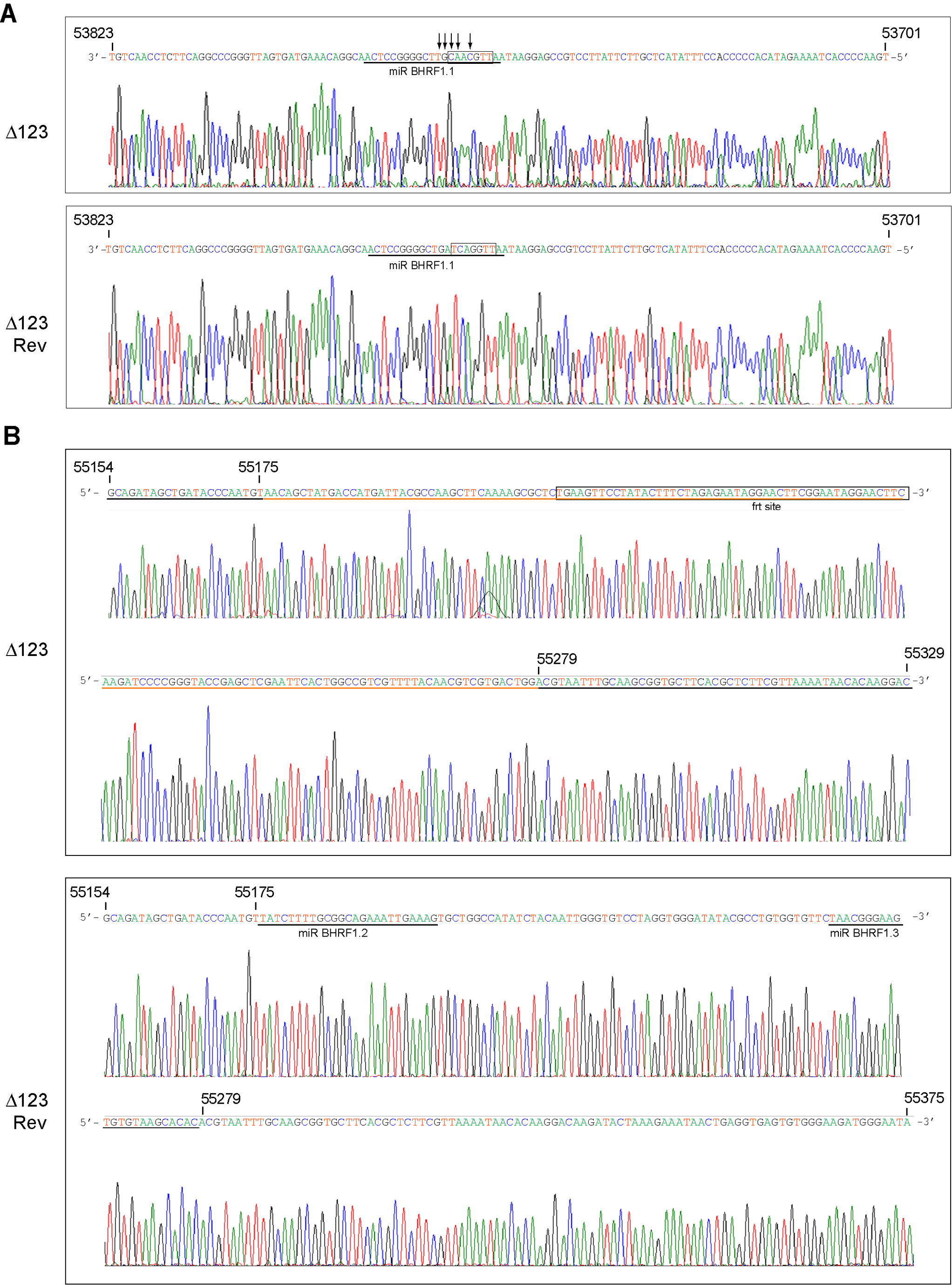

Supplement: Figure S2 — Electropherogram of sequenced miRNA regions in the Δ 123 mutant and Δ 123 revertant. (A) Shown is the sequenced lower strand spanning miR-BHRF1-1 (underlined) from B95.8 coordinates 53823 to 53701 (accession number V01555.2). The seed region of BHRF1-1 is boxed, the introduced mutations in the Δ 123 mutant (upper panel) are highlighted by arrows. The lower panel shows that the same DNA region in the Δ 123 revertant is identical to the wt miR-BHRF1-1 sequence. (B) DNA fragment spanning the BHRF1-2 and BHRF1-3 miRNAs. Upper panel: In the Δ 123 mutant, the BHRF1-2 and BHRF1-3 miRNAs are replaced by sequences from the kanamycin targeting plasmid (underlined in orange) including one frt site (boxed). EBV-specific sequences are underlined in black. Lower panel: Sequence spanning miR-BHRF1-2 and miR-BHRF1-3 (underlined) in the Δ 123 revertant shows perfect homology to EBV-wt sequences. B95.8 coordinates are indicated. (2.76 MB TIF) [file ppat.1001294.s002.tif]
